# A Case of Legionella Pneumonia Complicated by Pneumatoceles and Pneumothorax

**DOI:** 10.7759/cureus.98133

**Published:** 2025-11-30

**Authors:** Maako Shimizu, Yuichiro Shimoyama, Kentaro Ishikura, Kenji Motohashi, Yoshitaka Tomoda

**Affiliations:** 1 General Internal Medicine, Itabashi Chuo Medical Center, Tokyo, JPN

**Keywords:** legionella pneumonia, lung cyst, pneumatocele, pneumothorax ptx, recurrent pneumothorax

## Abstract

We report a rare case of *Legionella* pneumonia complicated by pneumatocele and recurrent pneumothorax. A previously healthy man in his 70s was diagnosed with *Legionella* pneumonia and treated with levofloxacin, resulting in initial improvement. However, he developed progressive hypoxemia with diffuse ground-glass opacities and traction bronchiectasis, requiring corticosteroid therapy. A 5-cm pneumatocele subsequently appeared in the right lower lobe, followed by two episodes of right-sided pneumothorax. Surgical bullectomy was performed to control the recurrence. The pneumatocele resolved spontaneously by discharge. Pneumatoceles are typically associated with necrotizing infections but have not been previously reported following *Legionella* pneumonia. This case underscores a previously undocumented structural complication and highlights the importance of radiological monitoring and surgical readiness in selected patients with post-*Legionella* respiratory deterioration.

## Introduction

*Legionella* pneumophila is a recognized cause of severe community-acquired pneumonia. While acute pulmonary manifestations are well described, delayed complications such as interstitial lung disease, organizing pneumonia, or cystic changes are rare [1]. Pneumatoceles, which are thin-walled, air-filled cysts, are typically associated with *Staphylococcus aureus* infection or trauma but are rarely reported with atypical pathogens such as *Legionella*. Secondary pneumothorax following cystic changes has been described in viral pneumonias, including COVID-19, but rarely in *Legionella* infection [2]. We present a case of *Legionella* pneumonia complicated by pneumatoceles and recurrent pneumothorax, underscoring the importance of radiological follow-up.

## Case presentation

A 72-year-old man with only hypertension and otherwise healthy presented with a five-day history of fever and progressive fatigue. On arrival, his body temperature was 39°C, and his oxygen saturation was 95% on 3 L via nasal cannula. Chest auscultation revealed coarse crackles in the right lung field. Laboratory findings were significant for leukocytosis (WBC 11,900/μL), hyponatremia (Na 127 mEq/L), and an elevated CRP level (30.74 mg/dL) (Table 1).

**Table 1:** Laboratory findings at the time of admission

Test	1st Admission	2nd Admission	Normal Range	Units
White blood cell count	11,900	7,400	3,300–8,600	μL
Hemoglobin	14.8	11.7	13.7–16.8	g/dL
Platelet	131,000	589,000	158,000–348,000	μL
Total bilirubin	1.6	0.7	0.3–1.2	IU/L
Aspartate aminotransferase	39	12	8–40	IU/L
Alanine aminotransferase	30	9	5–45	IU/L
Lactate dehydrogenase	164	196	124-222	IU/L
Creatinine	0.8	0.6	0.61–1.08	mg/dL
Blood urea nitrogen	13.4	9.7	8–23	mg/dL
Sodium	127	136	136–147	mEq/L
Potassium	4.2	4.4	3.5–5.0	mEq/L
Chloride	91	97	98–108	mEq/L
C-reactive protein	307.4	145.8	0–1.4	mg/L

**Figure 1:**
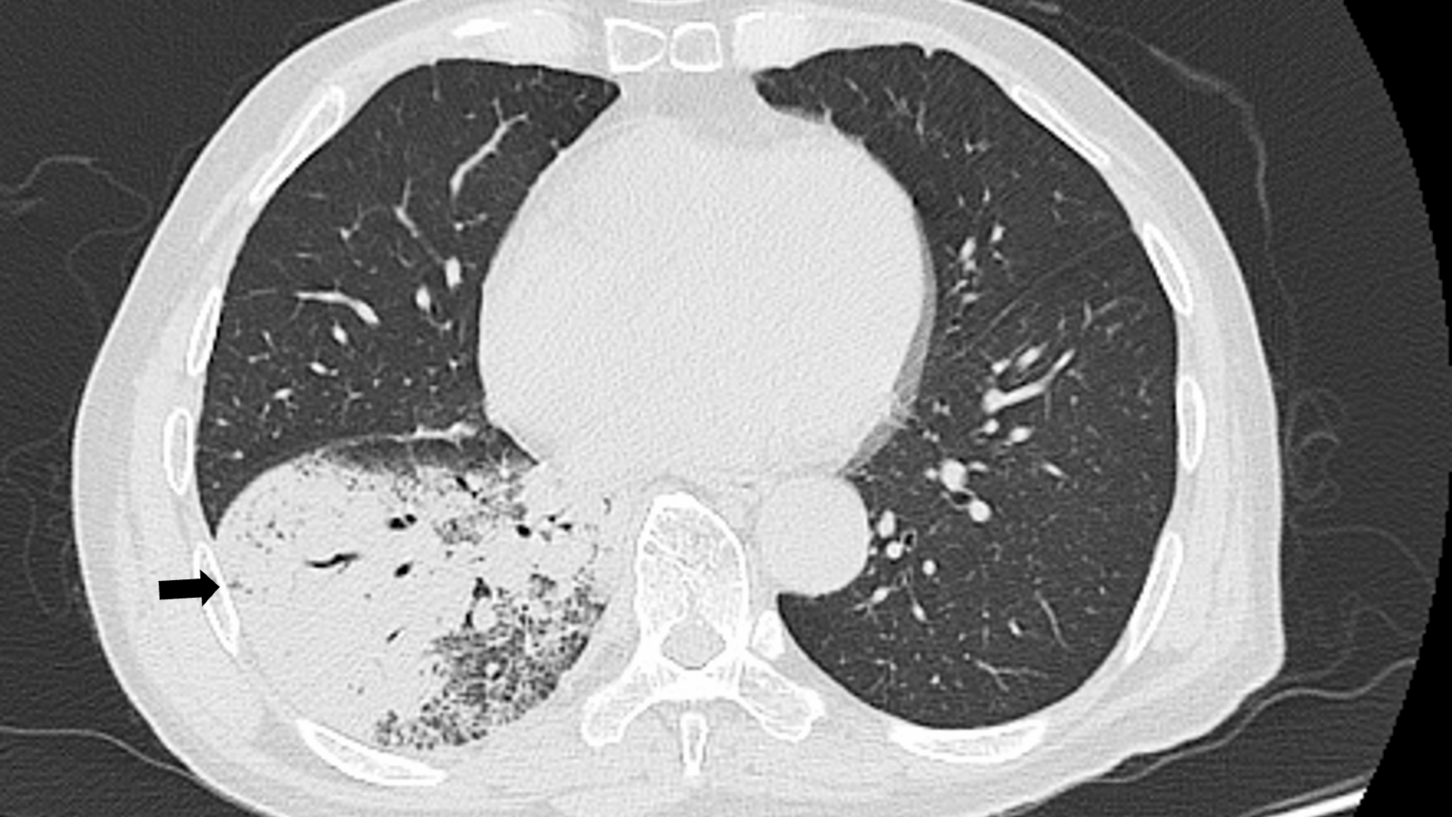
Chest CT on first admission showing consolidation with ground-glass opacities in the right lower lobe, consistent with Legionella pneumonia (arrow).

Chest computed tomography (CT) demonstrated consolidation with ground-glass opacities in the right lower lobe (Figure 1).

A urinary antigen test was positive for *Legionella*, confirming *Legionella* pneumonia. The patient was treated with intravenous levofloxacin, resulting in an initial improvement in pneumonia. However, respiratory failure persisted, requiring initiation of home oxygen therapy on day 21 of hospitalization.

**Figure 2:**
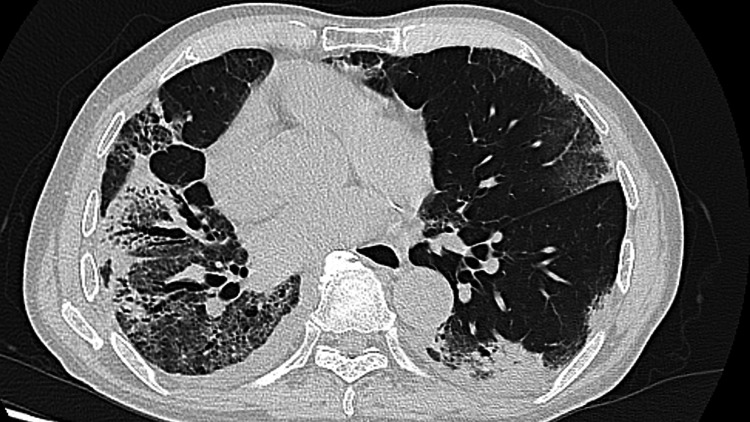
Chest CT on readmission showing diffuse bilateral ground-glass opacities with traction bronchiectasis.

Four days after discharge, he was readmitted due to worsening dyspnea. On admission, he presented with oxygen saturation of 95% while receiving 5 L/min of oxygen via face mask, indicating worsened hypoxemic respiratory failure. Chest CT revealed diffuse bilateral ground-glass opacities with traction bronchiectasis, suggestive of secondary interstitial involvement, most likely organizing pneumonia (Figure 2). Laboratory findings revealed elevated inflammatory markers and thrombocytosis (platelet count 58.9 × 10⁴/μL), the latter of which was considered to reflect a chronic inflammatory response following severe *Legionella* pneumonia (Table 1).

**Figure 3:**
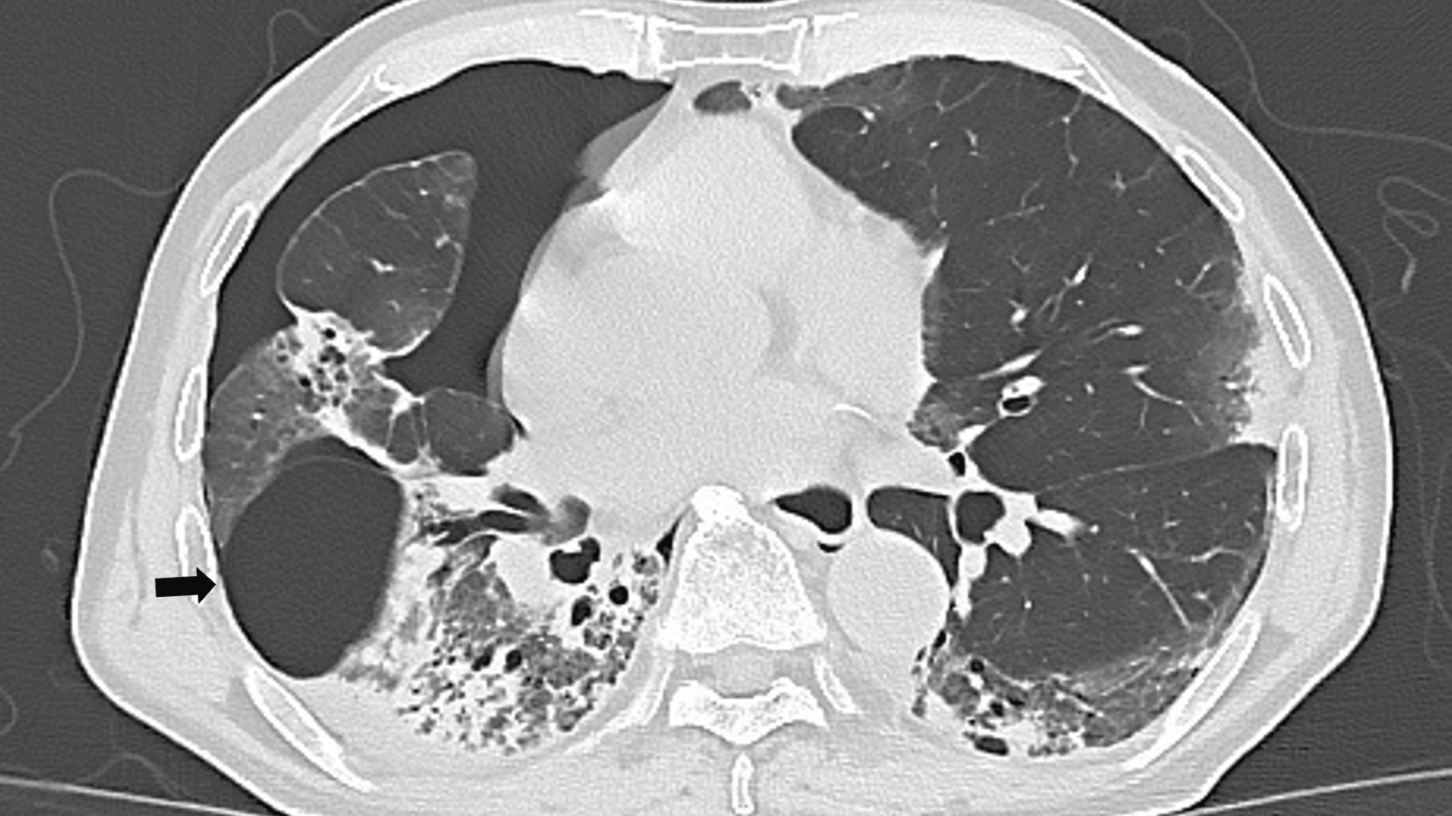
Chest CT on day five of readmission showing a right pneumothorax and a newly appeared 5-cm thin-walled, air-filled cavity in the right lower lobe, consistent with a pneumatocele (arrow).

**Figure 4:**
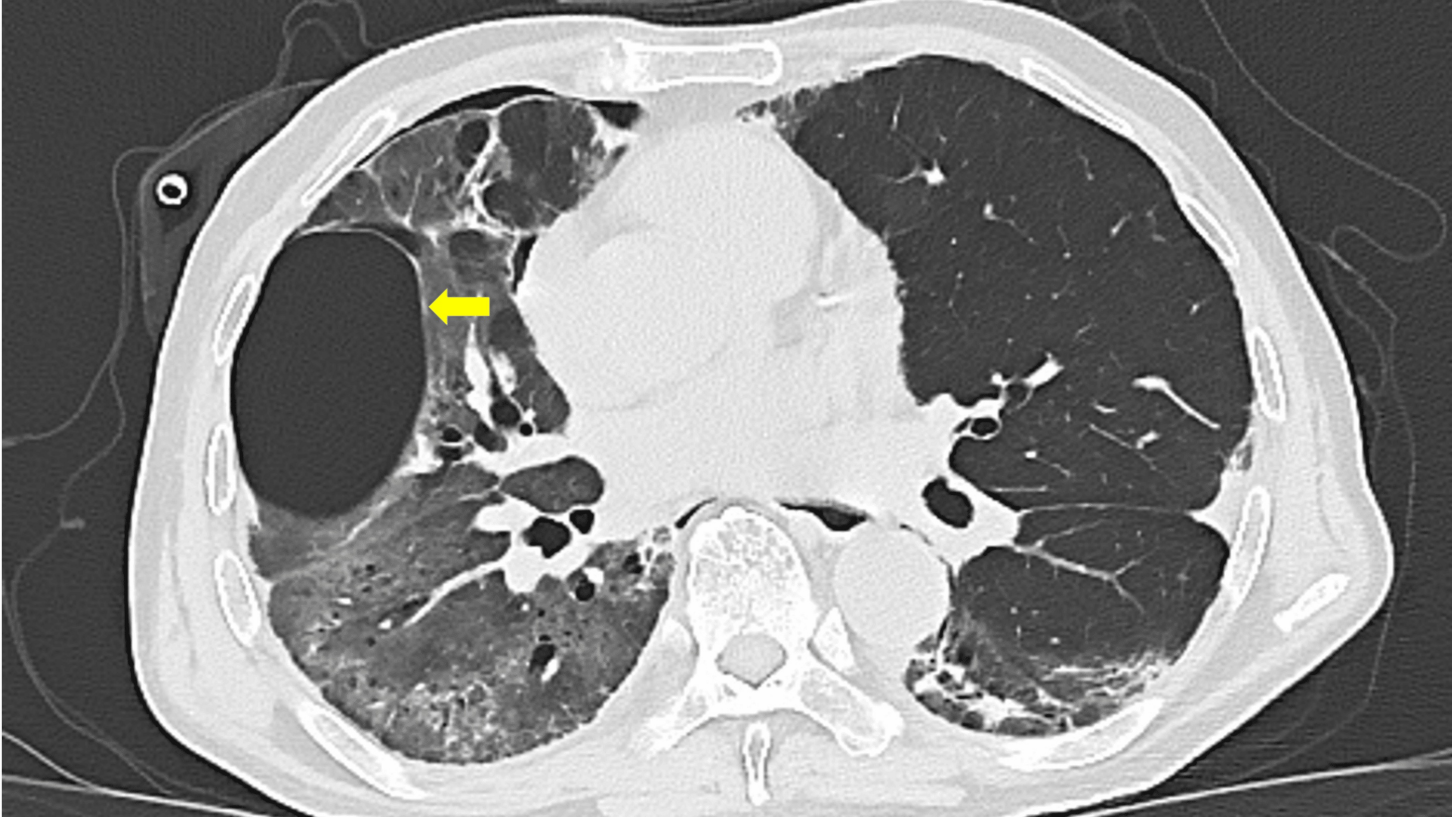
Follow-up chest CT after chest drainage showing a pneumatocele (arrow).

Blood cultures and sputum cultures were negative, making a new bacterial infection unlikely. Differential diagnoses at the time of respiratory decline included secondary bacterial infection, heart failure, acute respiratory distress syndrome (ARDS), and exacerbation of interstitial lung disease. Although bronchoscopy was considered for differential diagnosis, it was deferred due to the high risk associated with severe respiratory failure. Systemic corticosteroids (prednisolone 60 mg/day) were initiated based on clinical and radiological findings. On day five of readmission, a routine chest X-ray revealed a right-sided pneumothorax, and a chest drain was inserted. Follow-up CT revealed a right pneumothorax and a newly appeared 5-cm thin-walled, air-filled cavity in the right lower lobe, consistent with a pneumatocele (Figures 3, 4).

**Figure 5:**
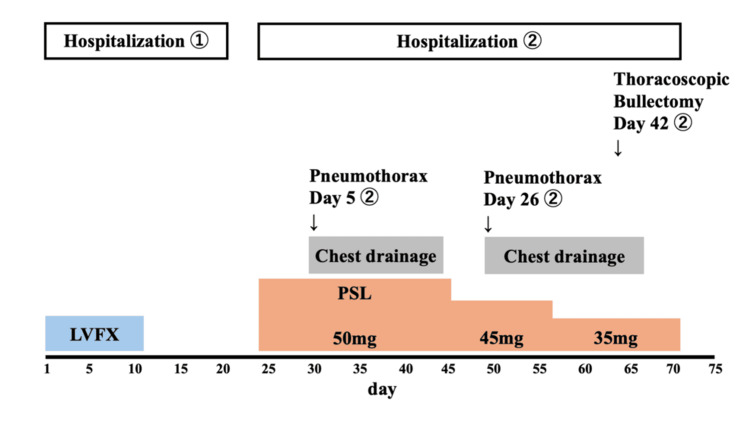
Clinical course of the first and second hospitalizations. LVFX, levofloxacin. PSL, prednisolone.

**Figure 6:**
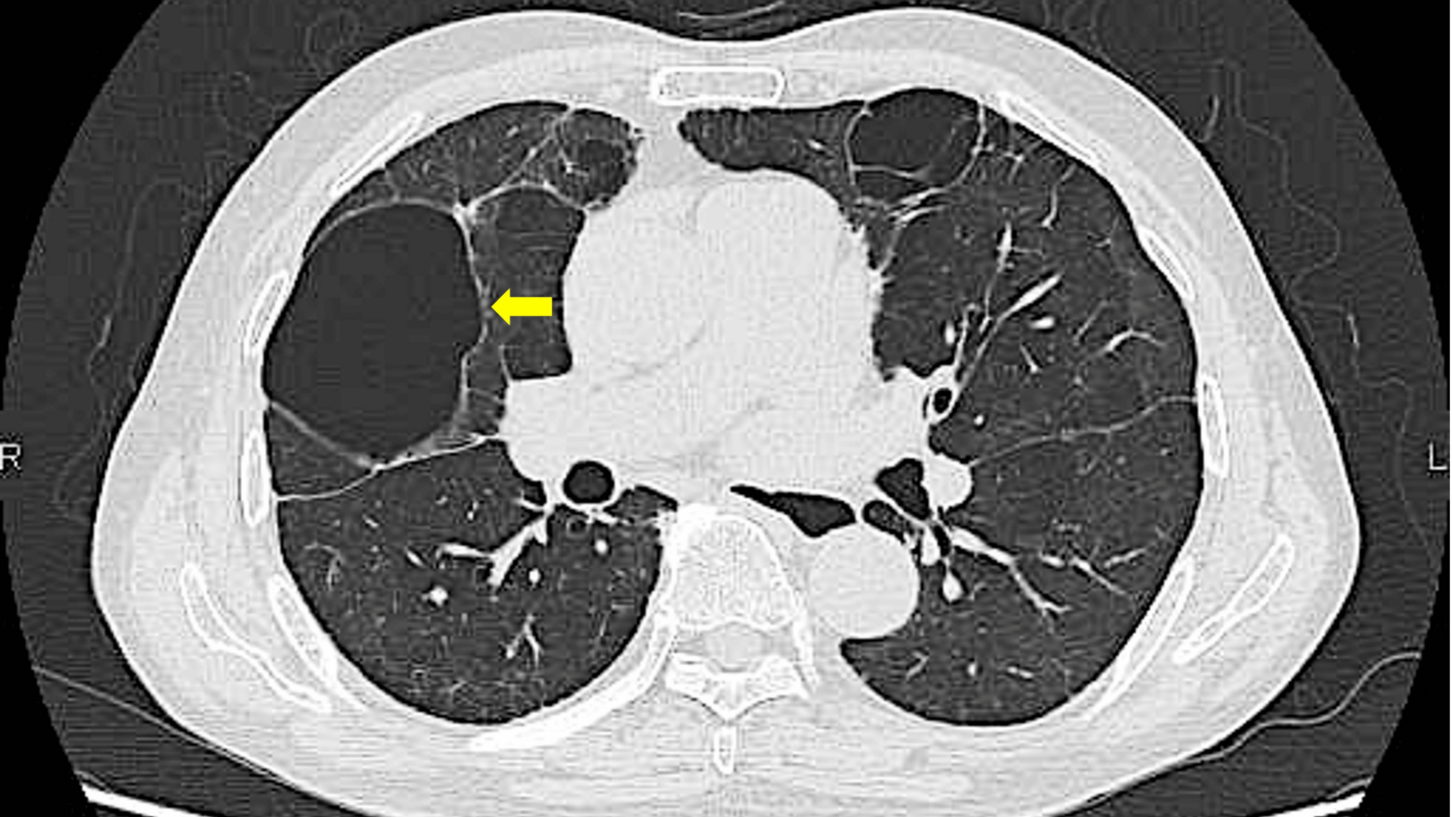
Chest CT scan six months after discharge showing the residual pneumatocele (arrow).

The pneumothorax resolved by chest drainage but recurred on day 26 of readmission. Surgical resection of a ruptured bulla in the right upper lobe was performed on day 42 of readmission. The presumed culprit lesion for pneumothorax was an apical bulla; however, the pneumatocele was also considered to have contributed to overall lung fragility. The cystic lesion remained stable in size on follow-up imaging by the time of discharge on day 47 of readmission (Figure 5). The steroid dose was gradually tapered every two weeks. During the six-month follow-up period after discharge, the pneumatocele remained stable without recurrence, and corticosteroids were eventually discontinued (Figure 6).

## Discussion

*Legionella* pneumonia typically presents with radiological features of airspace consolidation, often mixed with ground-glass opacities, and may be associated with pleural effusions or interstitial involvement. In this case, the initial chest CT demonstrated a mixture of dense consolidation and ground-glass opacities, consistent with prior reports of *Legionella* pneumonia imaging characteristics [2,3].

Pneumatoceles are thin-walled, air-filled cystic lesions that commonly develop in the context of necrotizing pneumonia, particularly in children with *Staphylococcus aureus* infections. In adults, they are relatively rare and typically associated with organisms such as *Streptococcus pneumoniae*, *Haemophilus influenzae*, *Klebsiella pneumoniae*, *Escherichia coli*, and COVID-19 [4,5]. The pathogenesis of pneumatocele formation is often attributed to a check-valve mechanism, in which inflammatory exudates or granulation tissue partially occlude small airways, allowing air to enter but not exit, leading to overinflation and cyst formation [6]. Other mechanisms may include necrosis of alveolar walls or bronchiolar obstruction due to inflammation or debris, especially in organizing pneumonia [7].

To our knowledge, there are no prior reports documenting pneumatocele formation following *Legionella* pneumonia. This case represents a novel manifestation, expanding the known spectrum of *Legionella*-related complications. Given the rarity of *Legionella*-induced pneumatoceles, the possibility of additional contributing factors should be considered.

One of the major complications of pneumatocele is pneumothorax, which may result from rupture of the cystic wall. This complication has been reported in association with various infectious etiologies and is particularly relevant in patients with pre-existing cystic lesions [8,9]. Pneumatoceles that are large or located subpleurally may predispose the patient to recurrent or refractory pneumothorax, as observed in our case. Although spontaneous pneumothorax has been reported in *Legionella* pneumonia, such instances are exceedingly rare [10,11].

In this case, the patient developed recurrent right-sided pneumothorax, ultimately requiring surgical intervention. The initial pneumatocele had formed in the right lower lobe but was not directly responsible for the pneumothorax; instead, bullous lesions in the apical region were identified intraoperatively as the source of air leakage. Nonetheless, the formation of pneumatocele and its temporal relation to steroid therapy raise questions about the role of corticosteroids in this pathological process.

Steroid-induced tissue fragility has been proposed as a contributing factor to pneumatocele and pneumothorax formation, particularly in patients with organizing pneumonia [7]. Granulation tissue within bronchiolar lumens may act as a one-way valve, facilitating cyst formation, while steroid therapy may delay tissue healing and increase susceptibility to alveolar rupture [7,12]. Furthermore, long-term corticosteroid use has been associated with an increased risk of secondary infections and impaired tissue repair, which may exacerbate the development and progression of pneumatoceles [7].

The limitations of this case include the absence of bronchoscopic evaluation, which was avoided due to the severity of respiratory failure at the time of readmission. Additionally, although the pneumatocele formed during corticosteroid treatment, it is not possible to establish a direct causal relationship. The histopathological correlation of the cystic lesion was also not obtained.

## Conclusions

In conclusion, pneumatocele and recurrent pneumothorax following *Legionella* pneumonia are extremely rare but clinically significant complications. In select cases, surgical intervention may be required. Clinicians should be aware of these potential sequelae, particularly in patients receiving corticosteroids or presenting with atypical radiologic progression.

This case highlights the potential for delayed cystic complications, including pneumatoceles and pneumothorax, following *Legionella* pneumonia. Vigilant radiological follow-up is essential to identify high-risk imaging features and to prevent life-threatening complications.

## References

[1] Blackmon JA, Harley RA, Hicklin MD, Chandler FW (1979). Pulmonary sequelae of acute Legionnaires' disease pneumonia. Ann Intern Med.

[2] Tan MJ, Tan JS, Hamor RH, File TM Jr, Breiman RF (2000). The radiologic manifestations of Legionnaire's disease. The Ohio Community-Based Pneumonia Incidence Study Group. Chest.

[3] Yu H, Higa F, Hibiya K (2010). Computed tomographic features of 23 sporadic cases with Legionella pneumophila pneumonia. Eur J Radiol.

[4] Camacho FA, Arevalo C, Connolly M, Modrak J (2024). Evolution of SARS-CoV-2 related pneumatoceles: a case report. Respir Med Case Rep.

[5] Puri MM, Srivastava A, Jain AK, Behera D (2011). Pneumatocele formation in adult Escherichia coli pneumonia. Ann Thorac Med.

[6] Quigley MJ, Fraser RS (1988). Pulmonary pneumatocele: pathology and pathogenesis. AJR Am J Roentgenol.

[7] Kadota T, Shimizu K, Tsurushige C (2012). Organizing pneumonia complicated by cyst and pneumothorax formation. Intern Med.

[8] Song Y, Jin J, Wang X, Zhang J, Li Z (2024). Recurrent spontaneous pneumothorax secondary to lung cystic lesions in a case of convalescent COVID-19: a case report and literature review. BMC Pulm Med.

[9] Colling J, Allaouchiche B, Floccard B, Pilleul F, Monneuse O, Tissot E (2005). Pneumatocele formation in adult Escherichia coli pneumonia revealed by pneumothorax. J Infect.

[10] Cunha BA, Pherez FM, Nouri Y (2008). Legionella community-acquired pneumonia (CAP) presenting with spontaneous bilateral pneumothoraces. Heart Lung.

[11] Chen YC, Lee CH (2017). Community-acquired Legionella pneumophilia pneumonia presenting with spontaneous pneumothorax. Kaohsiung J Med Sci.

[12] Ryerson CJ, Olsen SR, Carlsten C, Donagh C, Bilawich AM, Field SK, Churg A (2015). Fibrosing bronchiolitis evolving from infectious or inhalational acute bronchiolitis. A reversible lesion. Ann Am Thorac Soc.

